# Montage Matters: The Influence of Transcranial Alternating Current Stimulation on Human Physiological Tremor

**DOI:** 10.1016/j.brs.2014.11.003

**Published:** 2015

**Authors:** Arpan R. Mehta, Alek Pogosyan, Peter Brown, John-Stuart Brittain

**Affiliations:** Experimental Neurology Group, Division of Clinical Neurology, Nuffield Department of Clinical Neurosciences, Medical Sciences Division, University of Oxford, Level 6, West Wing, John Radcliffe Hospital, Oxford OX3 9DU, United Kingdom

**Keywords:** Transcranial electrical stimulation, Transcranial alternating current stimulation, Electrode montage, Photic stimulation, Physiological tremor, Entrainment, Phosphene, Current density modeling

## Abstract

**Background:**

Classically, studies adopting non-invasive transcranial electrical stimulation have placed greater importance on the position of the primary “stimulating” electrode than the secondary “reference” electrode. However, recent current density modeling suggests that ascribing a neutral role to the reference electrode may prove an inappropriate oversimplification.

**Hypothesis:**

We set out to test the hypothesis that the behavioral effects of transcranial electrical stimulation are critically dependent on the position of the return (“reference”) electrode.

**Methods:**

We examined the effect of transcranial alternating current stimulation (sinusoidal waveform with no direct current offset at a peak-to-peak amplitude of 2000 μA and a frequency matched to each participant's peak tremor frequency) on physiological tremor in a group of healthy volunteers (*N* = 12). We implemented a sham-controlled experimental protocol where the position of the stimulating electrode remained fixed, overlying primary motor cortex, whilst the position of the return electrode varied between two cephalic (fronto-orbital and contralateral primary motor cortex) and two extracephalic (ipsilateral and contralateral shoulder) locations. We additionally controlled for the role of phosphenes in influencing motor output by assessing the response of tremor to photic stimulation, through self-reported phosphene ratings.

**Results:**

Altering only the position of the return electrode had a profound behavioral effect: only the montage with extracephalic return contralateral to the primary stimulating electrode significantly entrained physiological tremor (15.9% ± 6.1% increase in phase stability, 1 S.E.M.). Photic stimulation also entrained tremor (11.7% ± 5.1% increase in phase stability). Furthermore, the effects of electrical stimulation are distinct from those produced from direct phosphene induction, in that the latter were only seen with the fronto-orbital montage that did not affect the tremor.

**Conclusion:**

The behavioral effects of transcranial alternating current stimulation appear to be critically dependent on the position of the reference electrode, highlighting the importance of electrode montage when designing experimental and therapeutic protocols.

## Introduction

Transcranial electrical stimulation (tES) is the umbrella term encompassing several non-invasive brain stimulation techniques that include direct current (tDCS), alternating current (tACS) and random noise (tRNS) stimulation [1]. tES is delivered by applying weak currents to the scalp that have been widely exploited to manipulate cortical excitability (tDCS, tRNS) or to interact with endogenous cortical rhythms (tACS). Even though the intended stimulation target is usually focal to a single cortical region, two electrodes are necessary to permit current flow. The second, so-called “reference” (or “return”) electrode, is typically positioned over an area presumed not to play an active role in the experimental paradigm [2], and its size sometimes made larger than the primary electrode with the intention of dissipating current at the return location [3].

However, modeling studies of current flow suggest that ascribing a neutral role to the return electrode may be a gross oversimplification (e.g., Ref. [4]). Indeed, keeping the position of the stimulating electrode over cortex invariant, whilst varying the position of the return electrode, has been shown not only to affect the electric field distribution across the entire cortex [5], but also the electric field distribution directly under the primary stimulating electrode [6]. The neuronal response to these imposed electric fields is itself non-trivial, with excitability affected by the orientation of dendrites relative to the electrical field gradients [7]. Additionally, stimulation is not confined to the cortical mantle, but pervades subcortical structures (e.g., Ref. [8]).

We set out to systematically and directly examine the behavioral implications of moving the return electrode by examining the effect of tACS on physiological tremor. This builds on earlier work that has shown that pathological [9] and physiological tremor [10], [11] provide a robust behavioral correlate of how tACS can modify oscillatory synchrony within the motor system. We adopted a sham-controlled experimental protocol where the stimulating electrode remained fixed overlying primary motor cortex, whilst the position of the return electrode varied between four positions, two cephalic and two extracephalic. Our experimental design also took into account the known differential effects of these electrode montages to generate phosphenes (the visual perception of flickering light) that might have otherwise confounded any observed entrainment effect.

## Materials and methods

### Participants

The study was performed on 12 healthy volunteers (9 males; mean age 26 years, range 19–36 years), all of whom provided informed written consent. All participants were right-handed. They were asked to refrain from ingesting any products with caffeine both during, and in the hour prior to, the study. The study was approved by the University of Oxford Central University Research Ethics Committee, in accordance with The Code of Ethics of the World Medical Association (Declaration of Helsinki) for experiments involving humans.

### Study design

The effect of rhythmic transcranial stimulation of the motor system on physiological postural tremor was studied using sham-controlled transcranial alternating current stimulation (tACS). tACS was applied at each participant's peak tremor frequency. Since the stimulation frequency was not forced to align with the ongoing tremor frequency, slow drifts in phase-alignment resulted between stimulation and tremor waveforms. Accordingly, this technique permits the online evaluation of phase stability (entrainment) and amplitude modulation as a function of the phase-alignment between the rhythmic tremor and stimulation signals [9], [10], [11], [12], [13].

**Figure 1:**
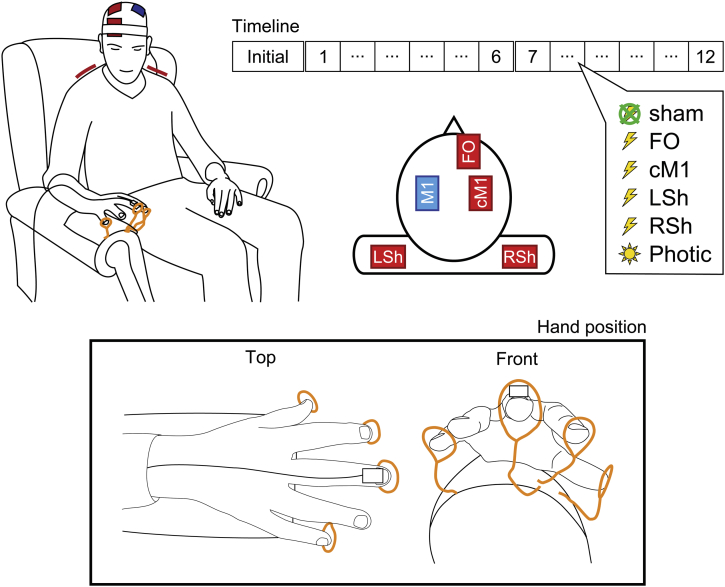
Experimental design depicting schematic illustrations of the tACS electrode montages and exemplar hand position adopted for recording of physiological postural tremor via accelerometry. The primary stimulating electrode was placed over left primary motor cortex, M1, along with four return electrode positions: fronto-orbital, FO; contralateral M1, cM1; left shoulder, LSh; and right shoulder, RSh. The Timeline shows the repeated measures sham-controlled study design; after an initial 360 s tremor recording to ascertain the participant's peak tremor frequency, the order of the six conditions (4 tACS conditions, photic stimulation, and sham condition) was randomized into two cycles of six 180 s experimental blocks, each separated by a 30 s rest period.

To address the principal question of whether the position of the return electrode significantly influences the effect of stimulation, we kept the primary stimulation site constant (left primary motor cortex, M1), whilst the return electrode was rotated between four possibilities: two cephalic positions – fronto-orbital (FO) and contralateral (right) primary motor cortex (cM1) – and two extracephalic positions – right and left shoulder (RSh and LSh, respectively; Fig. 1). These locations reflect the most common arrangements used by the tES motor community, and have been chosen to offer a broad range of expected current flow patterns. In particular, the cephalic positions have traditionally dominated motor tES studies, offering distinct current density distributions (e.g., Ref. [4]), whilst extracephalic positions have already proven effective in similar tremor paradigms [9], [10], [11].

To distinguish the effects of stimulation from the widely reported retino-cortical phenomenon of stimulation-induced phosphenes, and demonstrate that this visual perception may not be uniform across different electrode montages [1], [14], [15], external photic stimulation was recruited into the experimental design. This was used both as a reference against which participants would rate the intensity of their perceived tACS-induced phosphenes, and in the assessment of the direct effects of flicker-induced modulation on the entrainment and amplitude of tremor.

### Experimental procedure

Participants were seated in a comfortable chair with arm rests in a well-lit room and wore earplugs throughout the experiment to abate any auditory clicks associated with photic stimulation. They were instructed to rest their right forearm on the arm-rest and extend their unsupported wrist with their fingers splayed (Fig. 1). Such a posture provoked an often visible postural physiological tremor. Once a comfortable position was attained, circular guides, consisting of coiled copper wires, were aligned to the tips of the participant's index, middle, little finger and thumb, so as to constrain the position of the hand and improve reproducibility of the posture between experimental blocks. Participants practised moving their fingers in and out of this posture until they were satisfied that they could easily resume a consistent position. They were asked to maintain vigilance with their eyes open and directed at their splayed fingers to maintain their position. There were two cycles of six randomly interleaved experimental blocks. Each cycle consisted of four transcranial stimulation conditions (primary motor cortex stimulation with varying return electrode positions: FO, cM1, LSh, RSh), one sham condition and one photic stimulation condition. Accordingly, participants were presented with each condition twice, and the sham condition was therefore embedded twice at different points in the experimental paradigm. These two sham blocks did not differ (see Results section) and were averaged to provide a baseline. Each block lasted 180 s, with 30 s of rest between each block. The experiment was preceded by an ‘initial’ tremor recording of 360 s (with a 30 s break at 180 s) that was used only to determine the peak tremor frequency (see Timeline in Fig. 1). Participants were asked to report if they were experiencing fatigue, at which point longer rest periods were introduced, as necessary.

After the ‘initial’ tremor recording, participants were introduced to photic stimulation – brief pulses of light delivered at the determined peak tremor frequency – and instructed that after each experimental block they would be asked to rate their perception of phosphenes in reference to this photic stimulation (0 = absence of any perception of phosphenes; 10 = phosphenes perceived as intense as photic stimulation).

### Tremor recording

A tri-axial accelerometer (Twente Medical Systems International B.V., Oldenzaal, The Netherlands) was attached onto the dorsum of the middle finger of the right hand. The orientation of the accelerometer was fixed across participants, with the *z*-axis traversing the plane of maximal tremor amplitude perpendicular to the ground. The accelerometer signal was recorded using a 32-channel Porti7 amplifier (Twente Medical Systems International B.V.) and custom-built software sampled at 2048 Hz.

### Transcranial stimulation

Stimulation was carried out in accordance with current safety guidelines [16], [17]. Single pulse transcranial magnetic stimulation was delivered via a Magstim 200 stimulator (Magstim, Dyfed, UK) using a figure-of-eight coil applied to the scalp overlying left M1 to locate the motor hotspot that consistently evoked contralateral middle finger movement [18]. This spot approximately corresponds with position C3 of the international 10–20 system of electrode placement [19], [20].

tACS was delivered through conductive rubber electrodes (5 cm × 7 cm; EASYCAP GmbH, Herrsching, Germany) enclosed in saline-soaked sponges using a battery-driven stimulator (DC-STIMULATOR PLUS, neuroConn GmbH, Ilmenau, Germany). The stimulation electrode was centered over the left motor hotspot to overlie M1. The four return electrodes were centered as follows to create four montages ([5], Fig. 1):

i) FO – over the right supraorbital region to overlie Fp2 of the international 10–20 system of electrode placement [19];
ii) cM1 – over the primary motor cortex of the right cerebral hemisphere, mirroring the position of the stimulation electrode [5], thereby overlying, or close to, the position C4 of the international 10–20 system of electrode placement [19];
iii) LSh – over the left shoulder [10], [11], specifically the superior fibers of the trapezius muscle, and
iv) RSh – over the right shoulder [9], mirroring the left shoulder position.

The cephalic (i-ii) and extracephalic (iii-iv) electrodes were secured in place using Velcro straps and hypoallergenic dressing tape, respectively, at the beginning of the study, such that all electrodes remained *in situ* throughout the experiment. The setup was optimized to ensure that impedance, as measured by the stimulation device, was always below 10 kΩ. The frequency of the sinusoidal stimulation waveform was matched to each participant's peak tremor frequency to the nearest 0.1 Hz (as determined by a visual examination of the power spectrum of the first principal component of the accelerometer signal from the 360 s ‘initial’ tremor recording, assessed in Spike2, version 7.12b, Cambridge Electronic Design Ltd, Cambridge, UK). Stimulation was sinusoidal, delivered with no direct current offset, at a peak-to-peak amplitude of 2000 μA. Stimulation began with a 10 s ramp-up in current, followed by a ramp-down after a further 10 s in the sham condition using a randomly chosen return electrode. The applied current was recorded by placing a custom-built cable containing a 1 kΩ resistor in series with the output of the DC-STIMULATOR PLUS. By recording the potential difference across this resistor, a direct measure of current flow was attained. This signal was then passed through a Dual Channel Isolation Amplifier (Twente Medical Systems International B.V.) before being recorded, in conjunction with the accelerometer, using the 32-channel Porti7 amplifier.

### Photic stimulation

Photic stimulation consisted of repetitive flashes of light delivered at the same frequency as that used for tACS using a CPS10 Photic Stimulator (SLE Ltd., Croydon, UK). The flash input energy was set to 0.1 J using a full-face round photic lamp positioned 30 cm directly in front of the participant on a table. Since the participants were instructed to remain vigilant of their hand position throughout the experiment, the flashes were perceived as being in the peripheral field of their vision. This signal was recorded using a custom-built photodiode passed through the Dual Channel Isolation Amplifier before being recorded on the 32-channel Porti7 amplifier.

### Data analysis

#### Accelerometry

**Figure 2:**
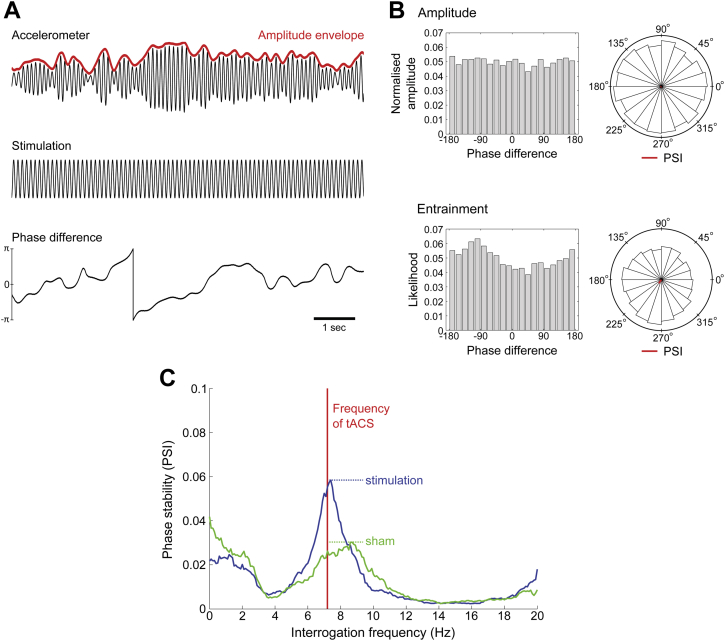
Analytical approach. (A) Exemplar filtered data of the first principal component of the tri-axial accelerometer tremor recording, tACS waveform, and computed phase-difference between the two signals. (B) Normalized amplitude and entrainment likelihood phase-difference histograms normalized as probability distributions, and corresponding angle histogram (polar) plots showing predominant phase preference (PSI vector colored in red). Phase stability (entrainment) is shown by the presence of a peak in the likelihood distribution. (C) Exemplar phase stability profile for a single participant for the RSh montage. Comparing the peak entrainment in the stimulation condition (blue line) with sham (green line; see Material and Methods section) as a percentage change in phase stability allows for robust quantification of the direct effect of stimulation on tremor oscillations. Such profiles also illustrate that tACS ‘pulls’ the frequency of oscillation toward the tACS frequency (vertical red line). Phase stability profiles for all participants for the RSh montage can be found in the Appendix A. (For interpretation of the references to color in this figure legend, the reader is referred to the web version of this article.)

Data were analyzed off-line using Matlab 8 (version R2013a, The MathWorks, Inc., Massachusetts, USA). Maximal tremor frequency was determined from the first principal component of the tri-axial accelerometer signal. Principal component analysis ensures that the plane of maximal tremor power is considered, accounting for any minor variations in the placement of the accelerometer or orientation of the hand between participants. The spectral peak was determined per experimental block using Thomson's multi-taper method [21], [22], using *K* = 12 tapers. The signal was then zero-phase bandpass filtered (forward-backward filtering) using separate third-order high- and low-pass Butterworth filters, centered about the peak tremor frequency for that block, affording a 2 Hz passband. Instantaneous phase and amplitude information were extracted from the filtered accelerometer (first principal component) and tACS waveforms via the Hilbert transformation [23]. The amplitude envelope of the derived accelerometer signal was variance stabilized per 180 s block using the Box–Cox transformation ([24], Fig. 2A).

Any entrainment effect of stimulation on tremor would imply adjustment of the physiological tremor rhythm towards stimulation over time, increasing the phase stability of the system. Phase stability relative to a reference signal (tACS in this case) can be assessed by first taking the time-dependent phase-difference ($\varphi_{t}$) between the accelerometer and stimulation time-series (Fig. 2A). Any preference in phase-difference (above that of chance) can be considered evidence of entrainment. To quantify the extent of entrainment, the phase synchronization index (PSI) between tremor and stimulation waveforms was computed for each 180 s block (Eq. (1)).

(1) $$PSI=\left|\sum_{t}e^{i\varphi_{t}}\right|$$

By construction, PSI = 0 if the signals are uncoupled and the phase-difference uniformly distributed, whereas PSI = 1 when the signals are perfectly synchronized, leading to a constant phase-difference. This can be visualized by constructing likelihood histograms, where we stratify phase-difference into 20 discrete bins (Fig. 2B).

By substituting multiple artificial stimulation signals at different frequencies for the tACS waveform, this approach can be extended to quantify the phase stability of tremor over a range of tremor frequencies (Fig. 2C). This approach accounts for any slight discrepancies that might exist between stimulation and tremor frequencies, whilst simultaneously assessing the frequency tuning characteristics of stimulation on the human motor system, as well as potential harmonic entrainment. Accordingly, phase stability profiles were constructed between 0 and 20 Hz in 0.1 Hz increments, applying 1 Hz smoothing regularization, for each stimulation block, and compared with analogously constructed profiles for the sham stimulation condition. This provided the typical entrainment values expected by chance in the absence of stimulation.

Since the tremor frequency can occasionally deviate from the chosen frequency of tACS in a manner that is not consistent with a stimulation-induced effect (see Results section), the maximum of the phase stability profile per block was averaged per condition (Fig. 2C), providing a single PSI value per participant, per condition. Note that assessment of the phase stability profile at only the applied stimulation frequency is analogous to our previous methodology [9], [10], [11].

The degree of amplitude modulation was similarly assessed by extracting the amplitude envelope from the Hilbert transform of the accelerometer signal (after Box–Cox transformation). PSIs were derived from the normalized (i.e., scaled as a probability distribution, such that ∑=1) amplitude histograms to quantify the degree of amplitude modulation (Fig. 2B).

#### Statistics

Statistical analyses were carried out using IBM SPSS Statistics for Windows (version 20.0.0, IBM Corp., New York, USA). Normality of data was examined using the Shapiro–Wilk test. One-way repeated measures analysis of variance (ANOVA) was used to examine the effects of Stimulation (5 levels: FO, LSh, RSh, cM1, and photic) separately on percentage change in phase stability (entrainment) and amplitude modulation. Sequence effects were examined with a one-way repeated measures ANOVA to assess for an effect of time (12 levels for each experimental block), and gross tremor characteristics of the sham condition (tremor amplitude, tremor frequency and sham entrainment) assessed between blocks using a separate repeated measures ANOVA, with fixed factor as ‘block’. Mauchly's test was performed to identify violations of the assumption of sphericity. Orthogonal planned comparisons to assess for the effects of the four tACS montages and photic stimulation on percentage change in phase stability (compared with sham) were performed by two-tailed one sample Student's *t*-tests. Note, that the use of planned comparisons may have inflated the chances of a Type I error, and so we also include the effect-size, stated as Cohen's *d* statistic. The Wilcoxon signed-ranks test was used to examine the self-reported propensity of each of the four tACS montages (plus sham) to provoke the perception of phosphenes (ranking 0–10). Correction for multiple comparisons was performed by adjusting *P* values for the false discovery rate (FDR). The significance level was set at *P* < 0.05. Unless otherwise stated, arithmetic means are reported ±1 standard error of the mean.

### Current density modeling

To assess whether the likely current density distribution induced by transcranial electrical stimulation might account for our observed behavioral differences, we additionally modeled the expected current density using a representative realistic head model derived from a single-subject MRI scan.

#### MRI segmentation

A structural T1-weighted MRI scan was acquired at a resolution of 1 × 1 × 1 mm^3^. Tissue segmentation was performed in a semi-automated fashion using a combination of the Functional Magnetic Resonance Imaging of the Brain (FMRIB, University of Oxford) Software Library (FSL; [25]), specifically the Brain Extraction Tool (BET; [26]), including skull and scalp extraction ([27]), FMRIB's Automated Segmentation Tool (FAST; [28]), and Seg3D: Volumetric Image Segmentation and Visualization, Scientific Computing and Imaging Institute (SCI Institute, University of Utah; [29]). Surface renderings of the major tissue types (skin, bone, gray matter, and white matter) are displayed in Appendix B (Fig. B.1), together with a sagittal projection of the assigned tissue types (Fig. B.2).

#### Isotropic conductivities

Stimulation pads affixed to the surface of the skin were modeled using custom-written code in Matlab, consisting of a 2 mm layer (simulated as saline) below an electrode layer. Electrode positions are depicted in Appendix B (Fig. B.1). Isotropic conductivities were set as in Dannhauer et al. (2012): skin (0.43 S/m), bone (0.0064 S/m), gray matter (0.33 S/m), white matter (0.142 S/m), cerebrospinal fluid (1.79 S/m), saline (0.367 S/m), and air (0.0001 S/m) [30]. All remaining tissues, largely composing of muscle and fat, were set to their average conductivities (0.08 S/m), as derived from Haueisen et al. (1997) [31].

#### Current density computation

We determined current density by first solving the Laplace equation for electric potential, φ, using the FEM solver provided in SciRun (SCI Institute, [32]; see Ref. [31]),

∇·(σ∇φ)=0

with σ as the tissue conductivities. The electric field distribution (E=−∇φ) and current density (J=σE) follow, where we chose to plot field strength, |J|, as our scalar measure of current density. For each montage arrangement, the primary stimulating electrode (overlying left M1) was set to a voltage level of +1 V. Current densities were then computed with each return electrode set to −1 V in turn. To facilitate comparison with previous studies, we scaled our steady-state potential maps, φ, to simulate a 1 mA current source, assuming a (typical) 10 kΩ load. The actual alternation in potential difference induced by our alternating current stimulation would cause the current density to undulate in a system-wide manner. Our simulations may, therefore, be interpreted as revealing the expected distribution of current density throughout the brain.

## Results

All participants completed the experiments and there were no adverse effects following tACS or photic stimulation. The mean peak frequency in the power spectra of physiological postural tremor was 8.28Hz ± 0.44 Hz and the application of tACS with the primary stimulating electrode overlying contralateral M1 was not associated with a consistent shift in peak tremor frequency (all |t_11_| ≤ 1.73, *P* > 0.05, two-tailed paired samples Student's *t*-tests).

## Rhythmic transcranial and photic stimulation entrain physiological tremor

Entrainment (phase stability) was quantified by calculating the percentage change of maximum PSI for stimulation (either via tACS or photic stimulation) compared with sham (see Materials and Methods section and Fig. 2C). This conformed to a Normal distribution at the group level (*P* > 0.05, Shapiro–Wilk test) and Mauchly's test did not show any violation of the assumption of sphericity. A one-way repeated measures ANOVA showed that phase stability differed between the different types of stimulation (*F*(4,44) = 3.38, *P* = 0.017).

The extent of amplitude modulation was similarly examined; this data also conformed to a Normal distribution at the group level (*P* > 0.05, Shapiro–Wilk test) and Mauchly's test did not show any violation of the assumption of sphericity. A one-way repeated measures ANOVA showed no significant differences in amplitude modulation between the different types of stimulation (*F*(4,44) = 0.103, *P* = 0.981). Thus, there was no significant modulation of physiological tremor amplitude by either tACS or photic stimulation.

To assess whether the randomization of the order of the four montages and photic stimulation between participants had been successful, one-way repeated measures ANOVAs were performed to assess the influence of time over the twelve experimental blocks. These confirmed that there was no sequence effect of experimental block on either entrainment (*F*(11,121) = 1.64, *P* = 0.096) or amplitude modulation (*F*(11,121) = 0.228, *P* = 0.995). This suggests that there were no significant after-effects provoked by a particular type of stimulation that might have interfered with the results. Moreover, there were no systematic changes observed in the sham condition between the first and second block in tremor amplitude (*F*(1,11) = 0.721, *P* = 0.414), tremor frequency (*F*(1,11) = 1.947, *P* = 0.190) or sham entrainment (*F*(1,11) = 1.644, *P* = 0.226).

### tACS montage differentially influences the extent of tremor entrainment and phosphene generation

**Figure 3:**
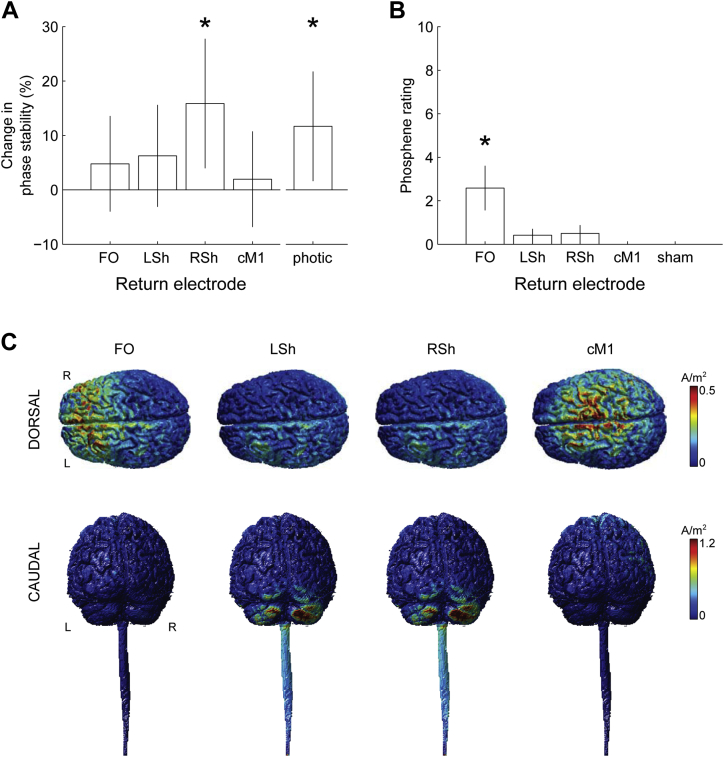
Group behavioral results and current density modeling. (A) Bar chart of percentage change in phase stability with respect to type of stimulation (tACS primary electrode fixed over left primary motor cortex, M1, with four different positions for the return electrodes: fronto-orbital, FO; contralateral M1, cM1; left shoulder, LSh; and right shoulder, RSh, *versus* photic stimulation). The ordinate reflects the pairwise percentage change in maximal PSI in the stimulation condition relative to sham (see Material and Methods section and Fig. 2C). The vertical error bars span the 95% confidence intervals for the groups. ∗ denotes significant results (RSh: t_11_ = 2.61, *P* = 0.024; Photic: t_11_ = 2.27, *P* = 0.044). (B) Bar chart of participant-reported phosphene ratings (0–10, where 0 = absence of any perception; 10 = as intense as the external photic stimulation) for each of the four tACS montages and for the no stimulation condition. ∗ denotes significant result (FO: *U* = 66, *P* = 0.005). (C) Simulated current density gray and white matter surface plots for each montage. L: Left, R: Right.

Planned contrasts using two-tailed one sample Student's *t*-tests revealed an effect of M1 stimulation on the phase stability of physiological tremor, but only when the stimulation was applied using the right shoulder return electrode montage (RSh: t_11_ = 2.61, *P* = 0.024, *d* = 0.754; LSh: t_11_ = 1.31, *P* = 0.218, *d* = 0.378; cM1: t_11_ = 0.437, *P* = 0.671, *d* = 0.126; FO: t_11_ = 1.06, *P* = 0.309, *d* = 0.308; Fig. 3A). Indeed, tACS over M1 with the return electrode over the right shoulder increased the phase stability of physiological tremor by 15.9% ± 6.1%, and, by simply changing the position of the return electrode, this effect was diminished. Phase stability profiles for all the participants for the RSh montage can be found in Appendix A.

The differential effect of the four electrode montages on each participant's perception of phosphenes was determined by comparing the reported phosphene rating scores, where 0 represented an absence of any phosphenes, and 10 represented a perception of phosphenes as intense as the external photic stimulation (Fig. 3B). Some perception of phosphenes was reported in three of the six return electrode positions (11/12 subjects reported phosphenes for FO, 6/12 for LSh, 6/12 for RSh, 0/12 for cM1, and 0/12 for sham). Taking intensity into account, the FO return electrode was the only montage that significantly induced phosphenes at the group level (Wilcoxon signed-ranks test, *U* = 66, *P* = 0.001, FDR-adjusted *P* = 0.005). Since the FO montage did not significantly influence tremor entrainment, we rule out the possibility that the observed stimulation-induced entrainment emerged as a secondary effect of phosphene perception.

**Figure 4:**
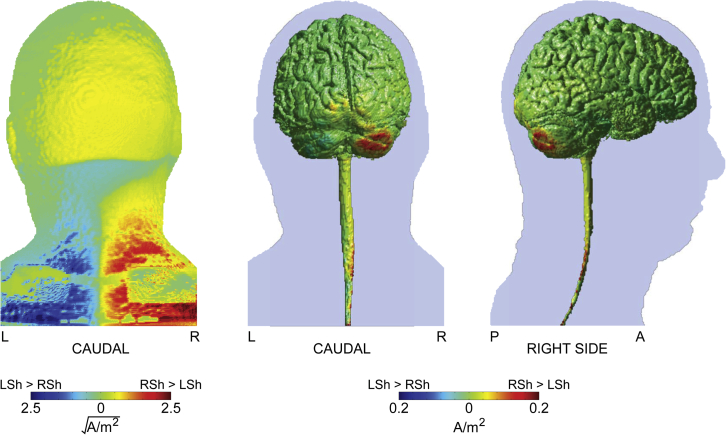
Modeling the influence of the position of the extracephalic return electrode on induced current density. Left panel: Caudal view of the simulated current density (square-root transformed) over the skin surface showing a broad increase in surface current density in the RSh *versus* the LSh configuration. Middle and right panels: Gray and white matter surface plots of the difference in simulated current density induced by RSh *versus* LSh return electrode montages. L: Left, R: Right, A: Anterior, P: Posterior.

Current density modeling shows that the greatest stimulation-induced current densities lie between the primary stimulating and return electrodes, with a tendency to focus around the cerebellar hemispheres in the case of extracephalic return locations (Fig. 3C, see also Appendix C.1). Contrasting the induced current density over the surface of the skin reveals a broad increase in current when employing a RSh return electrode when compared with a LSh return electrode (Fig. 4). This is associated with a marked increase in current penetration into the gray matter in the right cerebellar hemisphere (ipsilateral to the peripheral tremor, Fig. 4) and lower-cervical/thoracic spinal cord. Notably, the increase in simulated cerebellar current density was 7.2% higher in the right cerebellar hemisphere using a RSh return electrode than with a LSh return electrode. This difference is comparable with that empirically observed during stimulation, where a 9.0% relative increase in entrainment was observed for the RSh return electrode relative to the LSh return electrode.

### Photic stimulation entrains physiological tremor

The planned contrast using a two-tailed one sample Student's *t*-test revealed a significant effect of photic stimulation on the phase stability of physiological tremor (t_11_ = 2.27, *P* = 0.044, *d* = 0.656; Fig. 3). This effect was weaker (11.7% ± 5.1% increase in phase stability) than that provoked by tACS with the right shoulder return montage (15.9 ± 6.1%).

## Discussion

Our results suggest that rhythmic non-invasive electrical brain stimulation can influence activity in the human motor system, and that its ability to do so may be critically dependent on the chosen electrode montage. In our experimental paradigm, altering only the position of the return electrode (often termed the “reference” electrode) had a profound effect, such that only the right shoulder return significantly entrained physiological tremor. Furthermore, the effects of electrical stimulation are distinct from those produced from direct phosphene induction, in that the latter were only seen with a montage that did not directly affect tremor.

Our findings lend important physiological support to the emerging view from current density modeling studies that suggest that the position of the return electrode is an important determiner of the current flow path through the brain from the primary stimulating electrode [6]. This implies that standard tES may concurrently modulate multiple cortical, as well as subcortical, neural networks. The present findings are consistent with this: the extracephalic return electrode (right shoulder) contralateral to the primary stimulating electrode (overlying left primary motor cortex) produced the largest spread of current of the various montages tested and was therefore likely to recruit regions distant from the primary target site, including subcortical regions such as the basal ganglia, cerebellum and brainstem. Targeted non-invasive stimulation of the cerebellum, for example, may help to prise apart the relative contribution of these structures [33]. Indeed, we have previously shown that there are differential effects of moving the position of the primary stimulating electrode, from left primary motor cortex to contralateral cerebellum (using a fixed extracephalic return electrode) on the degree of entrainment of various types of physiological tremor [10], [11]. Taken together, these studies demonstrate that subcortical networks may play an important role in the emergence of oscillatory physiological tremor. It remains to be proven whether next-generation, high-definition stimulation montages, such as the 4 × 1 ring electrode configurations [34] that provide a focal, non-arbitrary position for the return electrode, are able to replicate the effects of standard setups, although we may hypothesize that they might not induce as strong a behavioral effect, given the increased focality and consequent reduction in the extent of simultaneously activated neural substrates. Of course, the cytoarchitecture of the underlying cortical (and subcortical) circuitry, along with current flow gradients, are also expected to play a profound role in stimulation-induced recruitment [34].

We can speculate about the reasons why the other electrode montages did not provoke significant entrainment of physiological tremor, whereas using a right shoulder return did. Further work combining brain stimulation, imaging and modeling techniques is needed to elucidate the influence of transcranial electrical stimulation on the human motor system. With the forehead return electrode, it is possible that a smaller portion of the current may enter the brain and a relatively larger current be bypassed along the skin between the electrodes [35]. Alongside current penetration, flow gradients can dramatically alter neuronal response to stimulation [34]. Meanwhile, the precise neural substrates responsible for our observed behavioral effect remain uncertain. Indeed, it seems equally likely that simultaneous activation (or inhibition) of multiple neural areas may be key. Previous research that examined different cephalic montages showed that the M1-fronto-orbital montage was optimal [5]. However, this study used tDCS and examined its after-effects on corticospinal excitability by comparing the amplitudes of motor-evoked potentials, and so offered an approach distinct from our own, which examines the online effects of rhythmic stimulation on entrainment of tremor [36]. The optimal site of stimulation is likely to be highly dependent on task demands. For instance, the M1-fronto-orbital montage has been successfully applied in tACS studies of motor behavior to facilitate or inhibit motor responses in a frequency-dependent fashion [37].

The present study also highlights a differential response between using a right *versus* left shoulder return electrode montage. A feasible explanation for the discrepancy is that in the ipsilateral case (left shoulder return electrode), greater current traverses the outside of the body compared with the contralateral case (right shoulder return electrode) [35], such that the current gradient is altered. Figure 4 illustrates the voltage gradient and current density for the right shoulder return montage compared with the left shoulder return montage, as determined from our model. Whilst the current densities induced by extracephalic return locations at first glance appear comparable (Fig. 3C), the contralateral shoulder induces greater current across the whole brain, in particular the cerebellar hemisphere ipsilateral to the side of the motor task (Fig. 4). It is interesting to note that in an earlier study using a different paradigm, we found significant entrainment using an ipsilateral left shoulder return electrode, suggesting that at least some of the current under that arrangement can penetrate task-specific motor circuitry [10], [11]. Why this effect was not observed in the present study can most likely be attributed to two key differences. First, the posture assumed in the present study is different, being more strictly controlled than that previously employed (see Fig. 1, also Materials and Methods section). As such, the postural exertions and state of tonic muscle activity are likely to be different. Second, we now employ a more conservative analytic method that can account for modest shifts in tremor frequency between conditions over time.

Importantly, stimulation intensities were kept constant for each montage; thus, we did not increase the stimulation intensity when using the extracephalic return electrodes. Previous work has suggested that the distance between the primary stimulating, and secondary return, electrode correlates negatively with the magnitude of their effect [35]. Our findings suggest a more complex relationship that allows for the recruitment of key, lower threshold, network regions distant to the primary site of stimulation.

Our experimental design also allowed us to parse the effects of brain stimulation on the motor network *versus* phosphene generation. There has been debate whether tACS-induced phosphenes originate cortically or are retinal phenomena (e.g., Ref. [38], [39]). Either way, phosphenes are an undesirable side-effect that can confound the interpretation of experiments adopting tACS [1], [15]. Our finding of significant entrainment in the right shoulder return montage that generated negligible phosphenes, compared with a lack of entrainment in the fronto-orbital return montage that generated the most phosphenes, provides strong support that the observed stimulation effects were not secondary to this visual perception; rather, that the mechanisms of physiological tremor entrainment and phosphene induction are distinct. We also demonstrated that external photic stimulation can significantly, albeit weakly, entrain physiological tremor. Photic stimulation at the frequencies adopted in the present study are well-known to induce phase-locked sinusoidal oscillations in the occipital EEG [40], which we suggest may spill-out through association areas into oscillations driving the motor system. Indeed, our finding dovetails with an earlier study that demonstrated that the sharpness of tuning of physiological finger tremor was increased by photic stimulation, suggesting entrainment [41]. In contrast, another study failed to show that the waveform of physiological hand tremor was phasically related to a repetitive photic stimulus [42]. Our analytical method to assess entrainment is likely more sensitive than that adopted in the latter study and thus was able to detect the partial entrainment effects reported here. Note also that photic stimulation can entrain frank motor responses in the condition of photic myoclonus [43], perhaps representing a pathological exaggeration of the effects uncovered here. Notwithstanding the clear morphological differences between electrically induced phosphenes and those arising from direct photic stimulation, we believe the failure of the former to entrain motor behavior was due to their weak relative perceived intensity. Photic stimulation, by design, elicited a visual perception rating of 10, whereas the most prominent phosphenes (observed under a fronto-orbital return electrode) elicited a mean rating of just 2.6, which we suggest was too weak to induce direct motor effects.

Several possible limitations of the study should be discussed. Our sample size was relatively small, and sensory percepts were different between conditions. However, the tACS manipulation used here differs from more prevalent tDCS paradigms, insofar as it relies upon the paired relationship between the alternating current stimulation waveform and the incumbent tremor rhythm. This makes the approach an ideal paradigm to examine the question of electrode placement, since the analysis is sensitive only to within-block fluctuations. On the other hand, the possibility that tACS may impose secondary effects, such as altered cortical excitability or plasticity, remains, and might reveal itself as data drift and order effects. To mitigate these effects as much as possible, we performed two sham blocks embedded within the experiment. These were averaged to create a baseline and did not differ in their characteristics.

## Conclusion

The behavioral effects of transcranial electrical stimulation appear to be critically dependent on the position of the return electrode. This merits careful consideration of electrode montage and resultant current flow in designing experimental and therapeutic protocols.
