# Supplementary material for: Montage Matters: The Influence of Transcranial Alternating Current Stimulation on Human Physiological Tremor
Source: Brain Stimul. 2015 Mar-Apr;8(2):260–8. doi: 10.1016/j.brs.2014.11.003 (PMC4319690; doi:10.1016/j.brs.2014.11.003)
Supplement: Appendix A [file mmc1.docx]

**
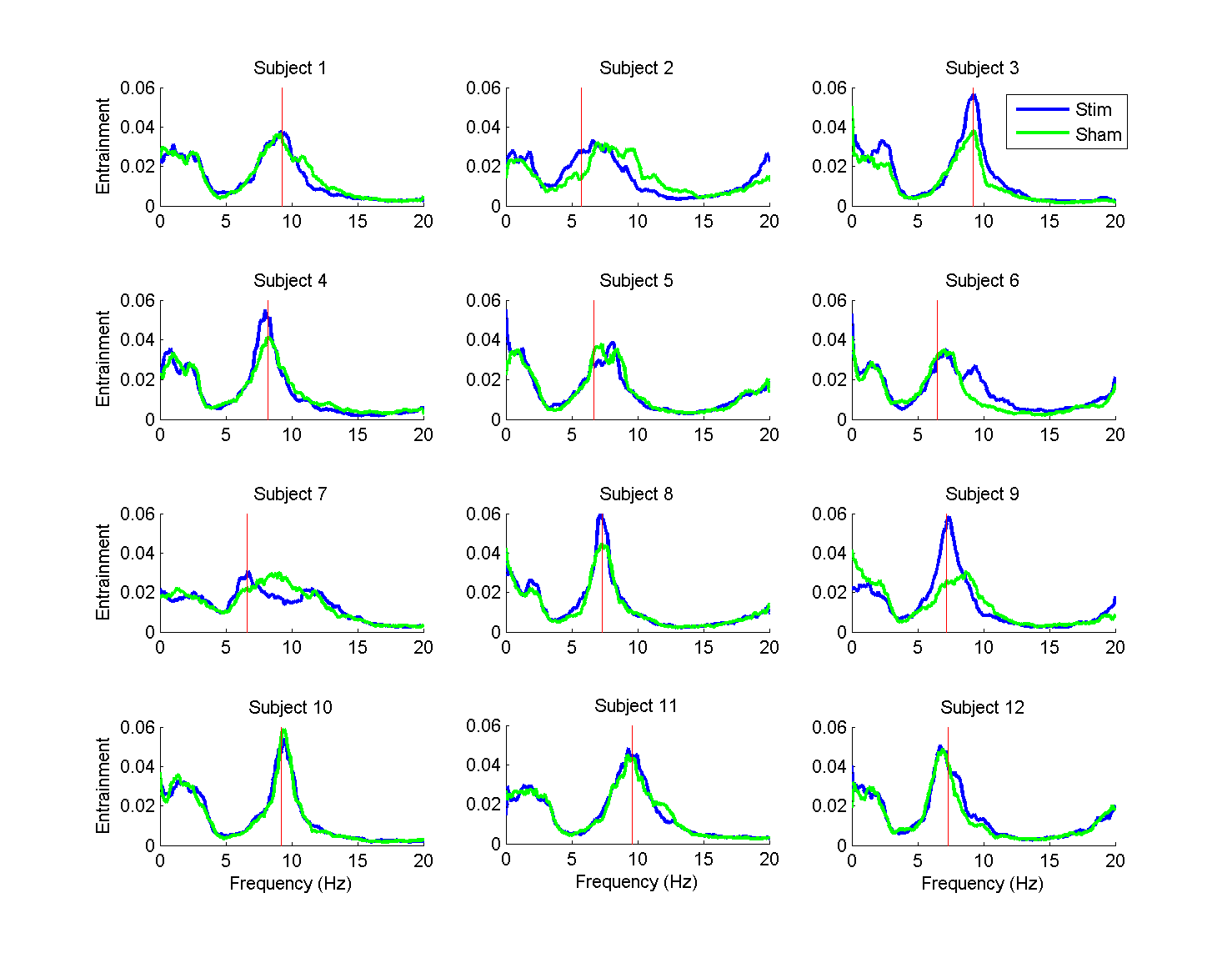
APPENDIX A – Phase stability profiles for RSh return electrode montage**

**Figure A.1**: Phase stability profiles for all twelve participants for the RSh return electrode montage allowing visualisation of the between-subject variability of the entrainment effect. Comparing the peak entrainment in the stimulation condition (blue line) with sham (green line; see **Material and Methods**) as a percentage change in phase stability allows for robust quantification of the direct effect of stimulation on tremor oscillations. Such profiles also illustrate that tACS tends to ‘pull’ the frequency of oscillation towards the tACS frequency (vertical red line).
