# Supplementary material for: Montage Matters: The Influence of Transcranial Alternating Current Stimulation on Human Physiological Tremor
Source: Brain Stimul. 2015 Mar-Apr;8(2):260–8. doi: 10.1016/j.brs.2014.11.003 (PMC4319690; doi:10.1016/j.brs.2014.11.003)
Supplement: Appendix B [file mmc2.docx]

**APPENDIX B - Current density modelling**


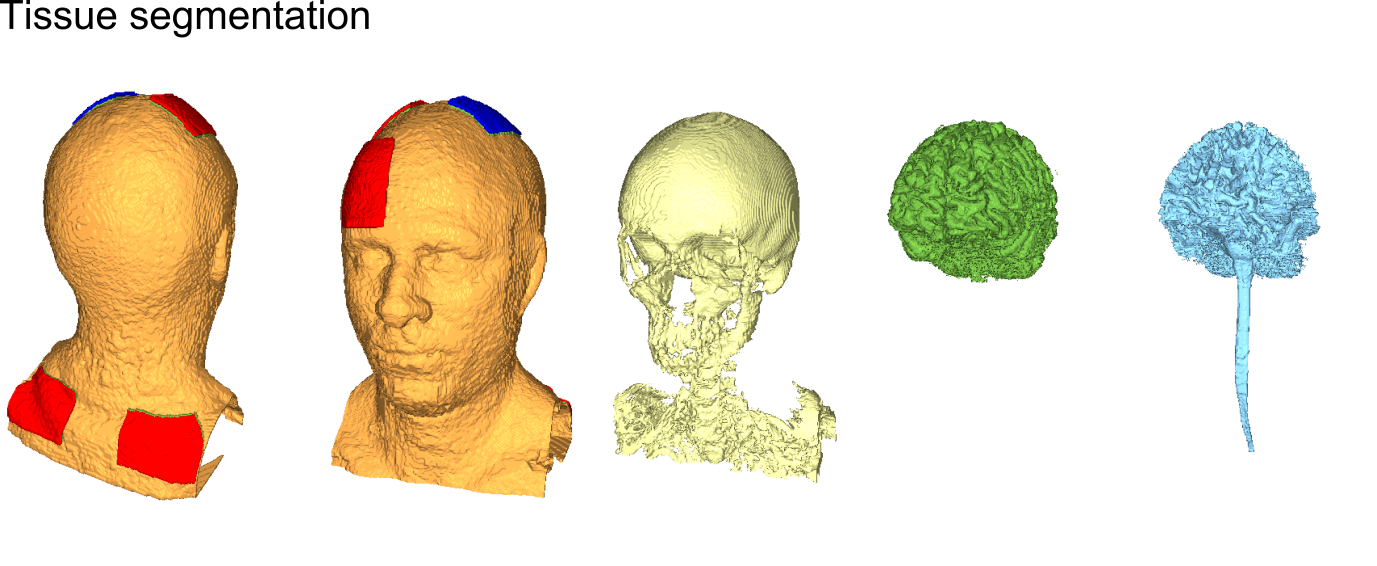


**Figure B.1**: Surface rendering of skin (showing fore and aft aspects to reveal electrode placement), skull, grey and white matter.


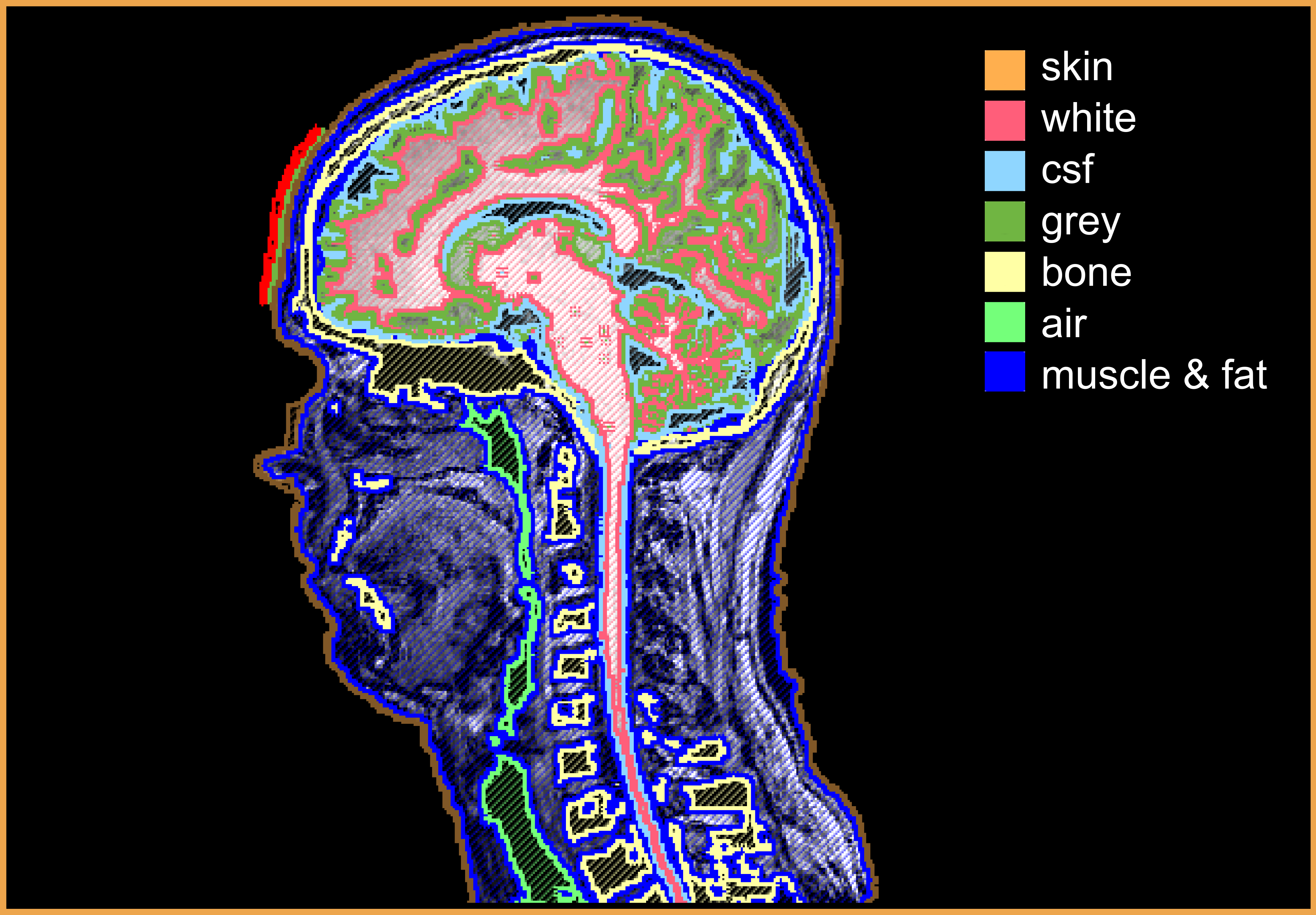


**Figure B.2:** Sagittal plane illustrating the segmentation of brain tissue used in our model.
