# Supplementary material for: Montage Matters: The Influence of Transcranial Alternating Current Stimulation on Human Physiological Tremor
Source: Brain Stimul. 2015 Mar-Apr;8(2):260–8. doi: 10.1016/j.brs.2014.11.003 (PMC4319690; doi:10.1016/j.brs.2014.11.003)
Supplement: Appendix C [file mmc3.docx]

**APPENDIX C – Subcortical current densities**

**
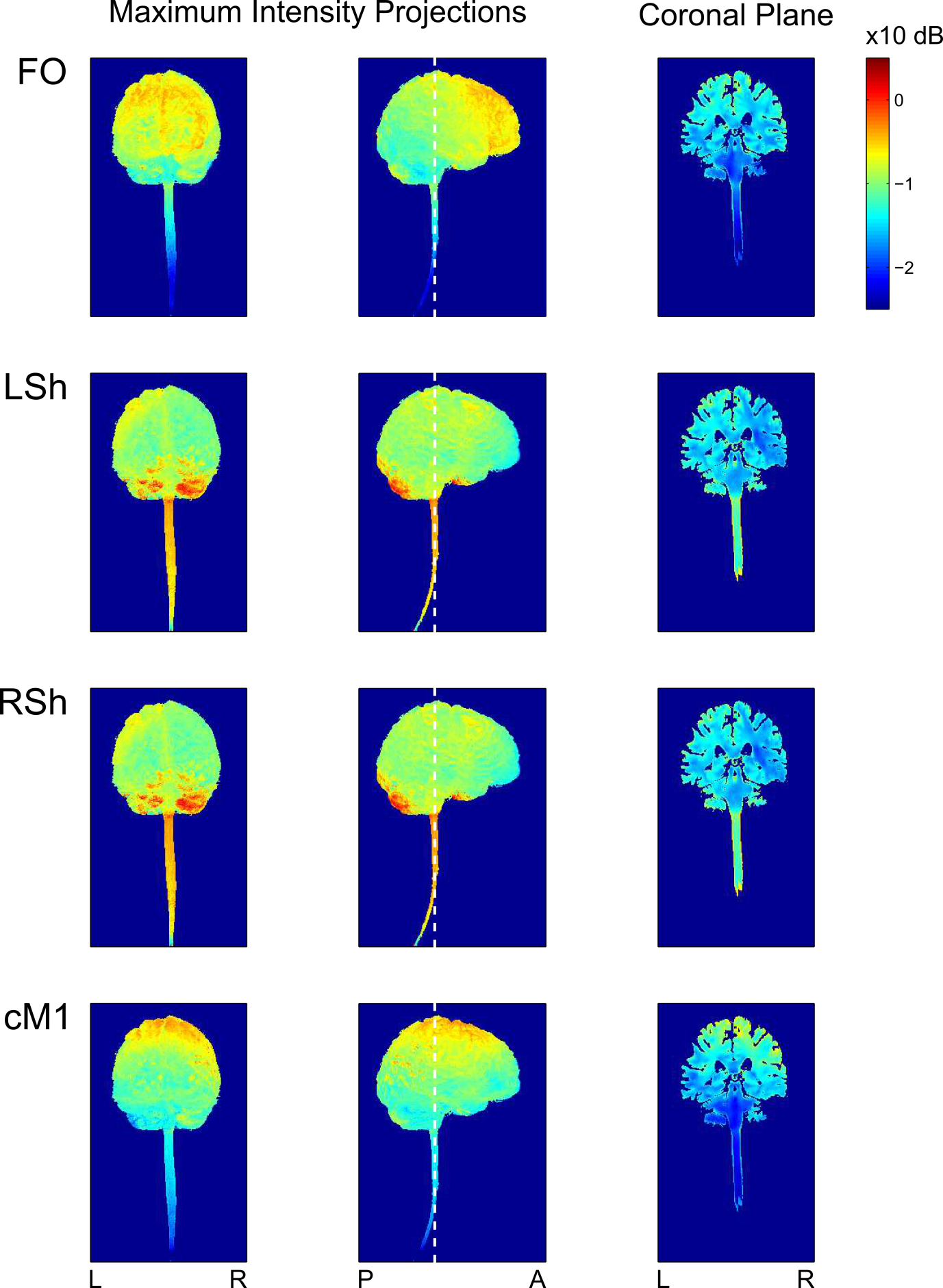
**

**Figure C.1**: Maximum intensity plots from a coronal and sagittal perspective depicting relative current densities (log_10_ A/m^2^) through cortical and subcortical regions. On the far right are coronal projection planes traversing the rostral portion of spinal cord. The dashed white line on the sagittal maximum intensity plot highlights the plane for the coronal image. Clearly, the greatest current density is induced at or about cerebral and cerebellar cortical surfaces. The spinal cord appears activated in the maximum intensity plots of LSh and RSh, though examination of the coronal plane suggests this is largely (but not exclusively) as a result of current flow through the surrounding cerebrospinal fluid.
